# First evaluation of genetic diversity and population structure of *Phelsuma inexpectata* (Gekkonidae), a critically endangered gecko endemic to Reunion Island

**DOI:** 10.1371/journal.pone.0338217

**Published:** 2025-12-12

**Authors:** Yann Gomard, Mickaël Sanchez, Margot Caubit, Markus A. Roesch, Alicia Bonanno, Johanna Clémencet

**Affiliations:** 1 UMR PVBMT (Peuplements Végétaux et Bioagresseurs en Milieu Tropical), Université de La Réunion, Saint-Pierre, La Réunion, France; 2 Association Nature Océan Indien, Petite-Île, La Réunion, France; 3 UMR PVBMT (Peuplements Végétaux et Bioagresseurs en Milieu Tropical), Université de La Réunion, Saint-Denis, La Réunion, France; State Museum of Natural History, GERMANY

## Abstract

The Manapany day gecko, *Phelsuma inexpectata*, is a critically endangered reptile endemic to Reunion Island (Southwestern Indian Ocean region). In the present study, we provide the first in-depth insights into the genetic diversity and population structure of the species across its main geographic range, limited to a narrow 14–km littoral fringe in the south of the island. We used two mitochondrial genes and twenty microsatellite loci to genotype 452 geckos sampled in anthropized and natural sites. Compared to other insular species of the *Phelsuma* genus, *P. inexpectata* displays a low genetic diversity with nine mitochondrial haplotypes detected, and based on the nuclear markers, a mean number of alleles (*N*_*a*_) of 2.8 ± 0.3, and an observed (*H*_*o*_) and expected heterozygosity (*H*_*e*_) reaching a maximum of 0.353 ± 0.053 and 0.345 ± 0.046 per site, respectively. For most sites, no significant deviations from Hardy–Weinberg equilibrium were detected. Along the limited distribution of *P. inexpectata,* isolation-by-distance patterns and geographical population structures were found with low first-generation migrants between sites. Genetic diversity distribution and structure are likely shaped by historical processes, including the fragmentation and isolation of relict populations, and anthropogenic-mediated colonization of novel habitats. The fine-scale population differentiation and genetic structuring, combined with the limited dispersal capacity of *P. inexpectata*, highlight the vulnerability of local gecko populations to extinction in the face of habitat fragmentation and loss. The low genetic diversity of *P. inexpectata* could limit its evolutionary potential and make it vulnerable to stochastic changes in its environment. Hence, efforts to conserve the genetic diversity should be strengthened, notably in natural sites harboring an original and remarkable genetic diversity.

## Introduction

Reptiles represent a large part of vertebrate biodiversity with currently more than 12,440 living species (1,260 genera and 94 families) making them the most diverse group of amniotic vertebrates [1]. Currently, reptiles are one of the most endangered vertebrate groups, with one in five species facing the threat of extinction [2,3]. The main threats include agriculture, logging, urban development, and invasive alien species [3,4]. In addition, based on data collected from 1970–2012, a global decline in reptile populations of 54–55% has been estimated [5], with significant declines predicted in the future due to climate change [6]. In this context, measures should be taken to protect reptiles, but there is a lack of data on their conservation status [7]. More knowledge should be acquired on species, including genetic and genomic data whose importance for the conservation and management of reptiles has been demonstrated in various studies [8], especially on threatened species such as tortoises [9–11], snakes [12], crocodiles [13,14], and lizards [15–20].

**Fig 1:**
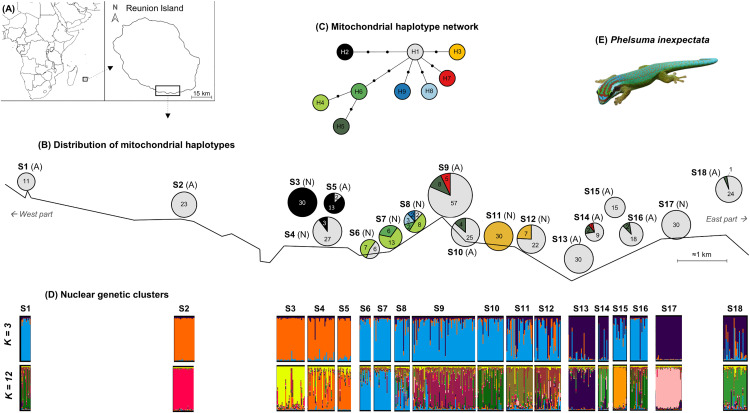
Location of study sites of *Phelsuma inexpectata.* **(A)** Location of Reunion Island in the Southwestern Indian Ocean region. **(B)** Location of the ten anthropized (“A”) and eight natural (“N”) sites. The pie charts show the distribution of the mitochondrial haplotypes at each site. The chart is colored according to the mitochondrial haplotypes detected and the size is proportional to the number of samples sequenced (N_total_ = 448). **(C)** Mitochondrial haplotype network based on the concatenated sequences of *cytb* and *12S* partial genes (1,190 bp). Each of the nine discovered haplotypes (H1 to H9) are indicated by one color. A dot on the link between haplotypes corresponds to one mutation. **(D)** STRUCTURE graphs with *K* = 3 and *K *= 12 genetic clusters based on the genotyping of 452 *P. inexpectata* at twenty microsatellite markers. Each color corresponds to one genetic cluster. Each specimen is represented by a single bar and colored according to the nuclear genetic cluster. **(E)** Photograph of an adult male *Phelsuma inexpectata* (picture: UMR PVBMT – Reunion University). Maps from (**A**) were realized by using the “worldHires” database from *mapdata* package [36] under the R program v4.2.1 [37]. On **(B)**, the coastlines are schematically represented for conservation purposes to protect spatial information associated with the species.

The Manapany day gecko, *Phelsuma inexpectata*, is an endemic reptile to Reunion Island, a French territory located 700 km east of Madagascar in the Southwestern Indian Ocean region (Fig 1). Classified as Critically Endangered on the IUCN Red List of Threatened Species, the species is threatened by habitat fragmentation and loss, anthropogenic activities, and invasive alien species [21]. Over the last decade, the abundance and area occupied by *P. inexpectata* in natural sites have declined [22,23]. Today, the remaining populations are restricted to a narrow 14–km littoral fringe in the south of the island (Fig 1A), and the total distribution area of the species, estimated at 24 ha, is small and highly fragmented [21,23]. In the context of habitat fragmentation and loss, the natural low dispersal capacity of *P. inexpectata*, estimated at a maximum dispersal distance of 100 m [24], could limit gene flow between isolated populations and increase the risk of local extinction. To conserve *P. inexpectata*, measures have been implemented and efforts have been made to acquire knowledge about the species [23,25–30]. Regarding the genetic knowledge of *P. inexpectata*, most available studies have addressed its phylogenetic relationships among the *Phelsuma* genus [31–34], and to date, the genetic diversity and population structure of *P. inexpectata* remain unstudied. Hence, in this study, we aimed to investigate the genetic diversity of *P. inexpectata* using two mitochondrial and twenty nuclear markers [35] and based on an extensive sampling across most of its distribution encompassing both natural and human-modified habitats (Fig 1B). In addition, we examined the population differentiation and structure, the presence of isolation-by-distance (IBD) patterns, gene flow through first-generation migrant analyses, and the potential occurrence of recent genetic bottlenecks. Given the extent of habitat fragmentation and loss along with the species’ limited dispersal capacity, a genetic structuring among sites is expected. Altogether, the generated data enhance our knowledge of *P. inexpectata* and, in addition to supporting ongoing management efforts, provide important guidance for the conservation of this threatened species.

## Materials and methods

### Ethic statement

The research was conducted with the permission delivered by the prefecture of Reunion Island (no DEAL/SEB/UBIO/2020-19). Captures, manipulations, and tissue sampling were approved by the Ethics Committee of Reunion Island for animal experiments (APAFIS #29467_2021012816034183_v2).

### Field sampling

Tissue samples were collected between March and September 2021 from 18 sites across the known geographical distribution of *P. inexpectata* along a narrow 14–km littoral fringe in the south of the island (Fig 1 and S1 Table). These sites represent the largest known areas containing the species and, along the coast, the sampling is nearly exhaustive [23]. Distances between neighboring sites ranged from 0.2 to 3.2 km, and the maximum distance between a pair of sites was 14.3 km. Each sampling area was considered as a specific site based on the presence of physical barriers (e.g., unfavorable habitats or roads) preventing potential natural dispersal of geckos coupled with the low dispersal capacity of *P. inexpectata*, estimated at a maximum of 100 m [24]. For each site, the type of habitat was defined as “natural” (sites characterized by the presence of remnant patches of pristine native vegetation for example: screwpine (*Pandanus utilis*) and Mauritius hemp (*Furcraea foetida*)) or “anthropized” (environments strongly modified by human activities, such as private gardens, urban parks or agricultural landscapes). Geckos were captured by hand or with small mammal traps [38]. Additionally, specimens originating at sites S6 and S7 from a captive head-starting program conducted by the local NGO Nature Océan Indien [39], were also sampled and included in the study. The sex of each specimen was determined based on morphological clues [40]. On the tail, at a point of autotomy, a small tip (< 1 cm) was collected using surgical scissors that were cleaned between cuts. Geckos were then released at the exact location where they had been captured. All collected tissues were preserved in 95% ethanol and stored at −20°C until DNA extraction. A subset of the collected samples (from sites S4 and S9) were previously used for the development and characterization of microsatellite markers for *P. inexpectata* [35].

### DNA extraction, mitochondrial marker sequencing and analyses

Total DNA was extracted from each sample using the NucleoSpin Tissue Kit (Macherey-Nagel). All samples were sequenced at two partial mitochondrial genes: cytochrome B (*cytb*) and *12s rRNA* (referred to herein as *12S*) already used for other gecko species in the region [16,31,33,34]. The primers used were CBL14753 (5’-TTC AAC TAC AAA AAC CTA ATG ACC C-3’) [31] and CBH15579 (5’-TGG GAT TGA TCG TAG GAT GGC GTA-3’) [41] for *cytb*, and 12Sa (5’-AAA CTG GGA TTA GAT ACC CCA CTA T-3’) and 12Sb (5’-GAG GGT GAC GGG CGG TGT GT-3’) [42] for *12S*. The amplification conditions were adapted from Harris et al. [43] and Rocha et al. [33]. All PCRs were performed in a 10 μL final reaction containing 1 U of Taq DNA polymerase, 0.24 mM of dNTPs, 10 pmol of each primer, 1X PCR Buffer, and 10 ng of DNA. The volume of MgCl_2_ in the PCR mix was 2.0 and 1.5 mM for *cytb* and *12S*, respectively. For *cytb*, PCR conditions consisted of an initial denaturation step at 94°C for 5 min, followed by 35 cycles of 94°C for 30 sec, 46°C for 40 sec, and 72°C for 45 sec, and a final elongation step at 72°C for 5 min. For *12S*, PCR conditions were an initial denaturation step at 94°C for 5 min, followed by 30 cycles of 93°C for 30 sec, 55°C for 1 min, and 72°C for 1 min, and a final elongation step at 72°C for 10 min. All PCR products were Sanger sequenced on both forward and reverse strands with the same primers used for amplifications. Each mitochondrial sequence was visually inspected and edited using Geneious Prime v2021.2.2 [44].

For each sampling site, for the concatenated mitochondrial genes (*cytb* and *12S*), the number of haplotypes (*H*) was determined, and the haplotype diversity (*hd*) and nucleotide diversity (*π*) were calculated using DnaSP v6.12.03 [45]. A haplotype network was constructed based on the concatenated mitochondrial genes using the *pegas* package [46] under the R program v4.2.1 [37]. A Wilcoxon rank test was used to examine the difference in *hd* between natural and anthropized sites.

### Microsatellite genotyping and analyses

Nuclear genotyping of the samples was performed using the twenty microsatellite markers developed for *P. inexpectata* and the associated protocols [35]. All PCR products were visualized on an ABI 3730XL DNA Analyzer using the GeneScan 500LIZ size standard (Applied Biosystems) and alleles were scored using Geneious Prime v2021.2.2 [44]. According to the microsatellite markers, 15–87 geckos were re-genotyped for verification or to remove any doubt in the scoring.

The software MICRO-CHECKER v2.2.3 [47] was used to test the presence of null alleles, large allele dropouts, and potential genotyping errors in each sampling site (10,000 randomizations). The GENEPOP v4.7.5 [48] was used to evaluate the linkage disequilibrium (LD) between all locus pairs for each of the 18 sites and the whole dataset (i.e., considering all sites as one unique site). For each locus, deviation from Hardy–Weinberg equilibrium (HWE) was tested in GENEPOP v4.7.5 with the Fisher’s exact test (10,000 dememorizations, 300 batches, and 5,000 iterations per batch). For both LD and HWE tests, the correction of Benjamini and Yekutieli [49,50] was applied for multiple comparisons. For each site, the genetic diversity was accessed by calculating the mean number of alleles (*N*_*a*_), the number of private alleles (*P*_*a*_, number of alleles detected only in a specific site), the observed heterozygosity (*H*_*o*_), and the expected heterozygosity (*H*_*e*_) using GenAlEx v6.51b2 [51,52]. Furthermore, deviation from HWE was tested in GENEPOP and the fixation index (*F*_*IS*_) was estimated in GENETIX v4.05.2 [53] using 1,000 bootstraps. Difference in nuclear genetic diversity between the type of sites (natural *vs.* anthropized) were tested with Wilcoxon rank tests on *H*_*o*_, *H*_*e*_, and *N*_*a*_.

### Population genetic differentiation

Genetic differentiation between all sites was evaluated by pairwise multilocus *F*_*ST*_ indices in the GENODIVE software [54], with 9,999 permutations and a Benjamini and Yekutieli correction. To visualize the genetic relationships between sites, a UPGMA (Unweighted Pair Group Method with Arithmetic Mean) hierarchical clustering method was applied to the *F*_*ST*_ matrix. The analysis was conducted assuming a constant molecular clock in the R software using the R package *ape* [55].

### Isolation by distance

The presence of isolation-by-distance (IBD) pattern was analyzed through the relation between genetic distance matrix (*F*/ (1- *F*_*ST*_)) against a geographic distance matrix between sites (km). Statistical significance was assessed by performing a Mantel test in GenAlEx using 9,999 permutations. The site S1, located at the extreme western end of the study area (Fig 1B), encompasses geckos which were considered as presumably recently introduced (Sanchez M., pers. comm.). Therefore, the IBD analysis was conducted excluding site S1 to avoid potential artifacts. IBD analyses were also performed on separate datasets containing anthropized (N_sites_ = 9) and natural sites (N_sites _= 8) only.

### Population clusters and structuration

The Bayesian clustering algorithm implemented in the software STRUCTURE v2.3.4 [56] was used to determine the number of genetic clusters based on the generated microsatellite data. The analysis consisted of 10 runs of 1,000,000 Markov Chain Monte Carlo (MCMC) iterations after 100,000 burn-in steps with *K* (number of genetic clusters) ranging from 1 to 20. The admixture model with correlated allele frequencies was used for the analyses without location prior (LOCPRIOR). Subsequently, the optimal *K* was determined using two methods, Evanno’s Δ*K* [57] and the mean log likelihood value of K (LnP(*K)*) implemented in the STRUCTURE HARVESTER online software [58]. The online CLUMPAK software [59] was used to examine the different runs and visualize the plots. In addition, a discriminant analysis of principal components (DAPC) was used as an alternative inference of the genetic structure of populations [60]. This analysis was performed using the R package *adegenet* [61] with Bayesian Information Criterion (BIC).

### Detection of first migrants

GENECLASS2 [62] was used to detect first-generation migrants between all investigated sites. We hypothesize that not all gecko sites were sampled and therefore used the L_home likelihood ratio [62,63]. The Bayesian method of Rannala and Mountain [64] was used. The Monte-Carlo resampling algorithm of Paetkau et al. [63] was used with 1,000 simulated individuals and a threshold probability of 0.01.

### Detection of bottlenecks

Recent reductions in effective population sizes were tested with BOTTLENECK v1.2.02 [65] using the Two-Phase Mutation Model with two multiple step mutation rates: a general vertebrate rate (p_g_ = 0.22) [66] and a reptile rate (p_g_ = 0.46) [67] as described in Buckland et al. [16]. The analyses were performed with 10,000 iterations, and statistical significance was assessed based on the results from one-tailed Wilcoxon signed-rank tests.

## Results

### Samples

A total of 452 individuals (198 females, 249 males, and 5 unsexed) were sampled from 18 sites: ten anthropized sites (S1, S2, S5, S9, S10, S13, S14, S15, S16, and S18) and eight natural sites (S3, S4, S6, S7, S8, S11, S12, and S17) (Fig 1B and S1 Table). The number of samples analyzed per site ranged from 11 to 71 (mean number of samples per site: 25.1).

### Mitochondrial genetic diversity

Amplification of mitochondrial *cytb* and *12S* partial genes yielded sequences with good quality for 448 out of the 452 samples. No deletions or insertions were detected after sequence cleaning. Sequence sizes were 804 bp and 386 bp for *cytb* and *12S*, respectively. A total of seven and three haplotypes were detected for the *cytb* and *12S* genes, respectively. Associated unique haplotypes were deposited in GenBank under the following accession numbers: OR126328-OR126334 and OR138116-OR138118 for *cytb* and *12S*, respectively. Concatenation of c*ytb* and *12S* sequences resulted in nine unique haplotypes of 1,190 bp: H1 to H9 (Fig 1C and Table 1). The number of haplotypes per site ranged from one to five, and seven sites had only one haplotype. Across the whole dataset, H1 was the most common haplotype (67.2%) followed by H2 (10.3%), H3 (8.3%), H4 (6.3%), H5 (3.8%), H6 (1.8%), H7 (1.3%), H8 (0.7%), and H9 (0.4%). H1 was detected from the western to the eastern part of the distribution, in 15 out of the 18 sampled sites, and was the only present haplotype in five sites. H5 was also widespread, from the center to the eastern part of the distribution, and was detected in half of the anthropized sites (Fig 1B). Except for H1, H5, and H7, which were detected in distant sites, the distribution of the other haplotypes was geographically structured. H2 was only detected in three geographically close sites (i.e., S3, S4, and S5), being the most abundant haplotype in two out of the three sites. H4 was detected only in the three geographically close natural sites S6, S7, and S8, while H6 was found only at sites S7 and S8. Similarly, H3 was detected only in the two geographically close natural sites S11 and S12. The only two private haplotypes (H8 and H9) were found in the same natural site S8. Overall, the most common haplotypes (H1 and H2) were found at both natural and anthropized sites. Only two unique haplotypes (H5 and H7) were found exclusively in anthropized sites, while five haplotypes (H3, H4, H6, H8, and H9) were unique to natural sites. Interestingly, the entire mitochondrial haplotype diversity was found along a geographical distribution within a distance of less than 5 km from sites S5 to S13. For all sites, nucleotide diversity (*π*) was low (0.000 to 0.002). Haplotype diversity (*hd*) ranged from 0.000 to 0.750 and 0.000 to 0.439 for natural and anthropized sites, respectively (Table 1). The haplotype diversity appeared to be twice as high in natural sites (mean *hd *= 0.289 ± 0.286) than in anthropized sites (mean *hd *= 0.154 ± 0.160) but this difference is not significant (Wilcoxon rank-sum test, p-value = 0.385).

**Table 1:** Genetic diversity at two mitochondrial and 20 microsatellite markers of *Phelsuma inexpectata* sampled at 18 sites on Reunion Island.

		Mitochondrial data (concatenated *cytb* and *12s*)				Microsatellite data					
Site	Habitat	*N*	*H*	*hd*	*π*	*N*	*N*_*a*_	*H*_*o*_	*H*_*e*_	*P*_*a*_	*FIS* [95% CI]
S1	A	11	1 (H1)	0.000	0.000	11	1.7 (± 0.2)	0.266 (± 0.064)	0.242 (± 0.054)	–	− 0.0522 [- 0.3277; 0.0755]
S2	A	23	1 (H1)	0.000	0.000	23	1.7 (± 0.2)	0.207 (± 0.053)	0.195 (± 0.049)	–	− 0.0835 [- 0.2940; 0.0712]
S3	N	30	1 (H2)	0.000	0.000	32	2.3 (± 0.2)	0.286 (± 0.054)	0.297 (± 0.053)	–	0.0494 [- 0.0732; 0.1327]
S4	N	30	2 (H1, H2)	0.186	< 0.001	30	2.3 (± 0.2)	0.353 (± 0.053)	0.345 (± 0.046)	–	0.0024 [- 0.1123; 0.0809]
S5	A	15	2 (H1, H2)	0.248	< 0.001	15	2.0 (± 0.2)	0.279 (± 0.061)	0.261 (± 0.048)	–	− 0.0461 [- 0.2036; 0.0044]
S6	N	13	2 (H1, H4)	0.539	0.001	13	2.1 (± 0.2)	0.270 (± 0.049)	0.274 (± 0.048)	–	0.0560 [- 0.1381; 0.1945]
S7	N	19	2 (H4, H6)	0.456	< 0.001	19	2.3 (± 0.2)	0.276 (± 0.048)	0.271 (± 0.048)	2	0.0348 [- 0.0940; 0.0892]
S8	N	17	5 (H1, H4, H6, H8*, H9*)	0.750	0.002	17	2.5 (± 0.2)	0.340 (± 0.044)	0.319 (± 0.041)	1	− 0.0393 [- 0.1828; 0.0314]
S9	A	70	3 (H1, H5, H7)	0.323	< 0.001	71	2.8 (± 0.3)	0.345 (± 0.051)	0.338 (± 0.048)	1	− 0.0122 [- 0.0754; 0.0359]
S10	A	29	2 (H1, H5)	0.246	< 0.001	29	2.4 (± 0.2)	0.253 (± 0.050)	0.247 (± 0.049)	1	0.0043 [- 0.1005; 0.0693]
S11	N	30	1 (H3)	0.000	0.000	30	2.4 (± 0.2)	0.324 (± 0.052)	0.319 (± 0.048)	–	0.0205 [- 0.0980; 0.0899]
S12	N	29	2 (H1, H3)	0.379	< 0.001	30	2.4 (± 0.2)	0.270 (± 0.045)	0.302 (± 0.050)	1	0.1463 [0.0190; 0.2243]
S13	A	30	1 (H1)	0.000	0.000	30	2.0 (± 0.2)	0.245 (± 0.051)	0.249 (± 0.047)	–	0.0378 [- 0.0935; 0.1275]
S14	A	12	3 (H1, H5, H7)	0.439	0.001	12	2.1 (± 0.2)	0.238 (± 0.054)	0.252 (± 0.050)	–	0.0710 [- 0.2053; 0.1925]
S15	A	15	1 (H1)	0.000	0.000	15	1.8 (± 0.2)	0.274 (± 0.062)	0.237 (± 0.052)	1	− 0.1084 [- 0.2923; 0.0088]
S16	A	20	2 (H1, H5)	0.200	< 0.001	20	2.2 (± 0.2)	0.253 (± 0.045)	0.271 (± 0.043)	–	0.0897 [- 0.0495; 0.1659]
S17	N	30	1 (H1)	0.000	0.000	30	1.9 (± 0.2)	0.258 (± 0.051)	0.252 (± 0.051)	–	0.0154 [- 0.1277; 0.1175]
S18	A	25	2 (H1, H5)	0.080	< 0.001	25	2.2 (± 0.2)	0.226 (± 0.044)	0.239 (± 0.048)	–	0.1000 [- 0.0602; 0.2023]

For each site, the type of habitat is indicated, A: anthropized; N: natural; *N* the number of genotyped specimens; *H* the number of haplotypes (H1–H9: haplotype designation, an asterisk indicates a private haplotype); *hd* haplotype diversity; *π* nucleotide diversity; *N*_*a*_ the mean number of alleles; *H*_*o*_ the observed heterozygosity; *H*_*e*_ the expected heterozygosity; *P*_*a*_ the number of private alleles; *F*_*IS*_ the fixation index [95% confidence interval].

### Nuclear genetic diversity

Nuclear genotypes were obtained for all 452 sampled geckos with 60% to 100% of amplified loci per specimen. For each microsatellite marker, the amplification rates ranged from 85.6% (Pinex_22) to 99.8% (Pinex_06 and Pinex_15) (S2 Table). Based on the whole dataset, all loci were polymorphic with at least two alleles detected per locus. These results are consistent with previous findings obtained at two sampling sites (i.e., S4 and S9) during the development of the nuclear markers [35]. A total of 75 distinct alleles were detected across all considered loci. For each locus, the total number of alleles *Na*_*tot*_, the observed *H*_*o*_ heterozygosity, and the expected *H*_*e*_ heterozygosity were low and ranged from 2 to 7, 0.023 to 0.517, and 0.022 to 0.520, respectively (S2 Table). All loci were in HWE. When all nuclear data are compiled as one unique site, LD was detected for three locus pairs (Pinex_72 and Pinex_44, Pinex_72 and Pinex_52, and Pinex_72 and Pinex_87) (all p-values < 0.001). However, analyses within each of the 18 sites separately revealed the presence of LD at site S3 only and for one locus pair: Pinex_07 and Pinex_34 (p-value < 0.05). Null alleles were detected for five microsatellite markers: Pinex_22 at site S4, Pinex_44 at site S13, Pinex_46 at sites S12 and S18, Pinex_61 at site S3, and Pinex_62 at site S6 (S2 Table). Given the detection of LD and null alleles in a limited number of sites, all loci were retained for subsequent analyses.

The mean number of alleles (*N*_*a*_) was low and ranged from 1.7 ± 0.2 to 2.8 ± 0.3 between the 18 sampled sites (Table 1). No significant difference was detected for *N*_*a*_ between the two types of sites with mean values of 2.3 ± 0.2 and 2.1 ± 0.3 for natural and anthropized sites, respectively (Wilcoxon rank- sum test, p-value = 0.108). Private alleles (*P*_*a*_) were detected in natural sites with two private alleles in site S7, and one private allele in sites S8 and S12. Private alleles were also detected in anthropized sites with one private allele found in each of the three following sites: S9, S10, and S15 (Table 1). The *H*_*o*_ and *H*_*e*_ ranged from 0.207 ± 0.053 to 0.353 ± 0.053 and from 0.195 ± 0.049 to 0.345 ± 0.046, respectively. Natural sites had significantly higher mean values of *H*_*o*_ (0.297 ± 0.037) and *H*_*e*_ (0.297 ± 0.031) than anthropized sites (mean *H*_*o *_= 0.259 ± 0.037 and mean *H*_*e *_= 0.253 ± 0.036) (Wilcoxon rank-sum tests, all p-values < 0.05). For all sites, no significant deviation from HWE was detected based on analyses done in GENEPOP. This is consistent with *F*_*IS*_ values obtained from GENETIX except for the site S12, at which a deficit is observed with an *F*_*IS*_ of 0.1463 and significantly different from zero (CI = [0.0190; 0.2243]).

### Population genetic differentiation and isolation by distance

All pairwise *F*_*ST*_ comparisons among sites were significant (all p-values < 0.05), with values ranging from 0.033 to 0.454 (Table 2). Overall, geographically close sites displayed low to moderate *F*_*ST*_ values (from 0.033 to 0.125), for instance, S3, S4, and S5; S6, S7, S8, and S9; or S11 and S12, with distances between sites ranging from 0.2 to 1.6 km (Table 2). In contrast, geographically distant sites had high *F*_*ST*_ values, such as sites S2 and S15, which are more than 8 km apart and display the highest *F*_*ST*_ value (0.454). Compared to all other sites, sites S2, S15, and S17 appeared the most differentiated, with all values of pairwise *F*_*ST*_ higher than or equal to 0.185 (Table 2). The UPGMA tree supported that sites generally clustered according to their geographic origins but also revealed that some geographically distant sites grouped together, exhibiting low genetic divergences as for instance the anthropized sites S1, S10, and S16 or S14 and S18 (*F*_*ST*_ values ≤ 0.064 and distances between sites ranging from 3.0 to 12.1 km) (S1 Fig and Table 2).

**Table 2:** Values of pairwise *F*_*ST*_ (lower diagonal) and distance in km (upper diagonal) between the 18 sampled sites for *Phelsuma inexpectata* on Reunion Island.

	S1	S2	S3	S4	S5	S6	S7	S8	S9	S10	S11	S12	S13	S14	S15	S16	S17	S18
**S1**	–	3.3	6.0	6.2	6.4	7.4	7.6	8.0	8.9	9.2	9.6	10.1	11.2	11.5	11.8	12.1	13.0	14.3
**S2**	**0.395**	–	2.7	2.9	3.1	4.2	4.3	4.8	5.6	5.9	6.3	6.9	7.9	8.2	8.6	8.9	9.7	11.1
**S3**	**0.221**	**0.195**	–	0.2	0.4	1.5	1.6	2.1	3.0	3.2	3.6	4.2	5.2	5.6	6.0	6.2	7.1	8.5
**S4**	**0.208**	**0.255**	**0.076**	–	0.3	1.2	1.4	1.9	2.8	3.0	3.4	4.0	5.0	5.3	5.7	6.0	6.8	8.3
**S5**	**0.233**	**0.365**	**0.125**	**0.065**	–	1.1	1.2	1.7	2.6	2.8	3.2	3.8	4.8	5.2	5.5	5.8	6.7	8.1
**S6**	**0.189**	**0.371**	**0.182**	**0.120**	**0.178**	–	0.2	0.8	1.6	1.8	2.2	2.7	3.7	4.1	4.5	4.8	5.6	7.1
**S7**	**0.192**	**0.348**	**0.167**	**0.140**	**0.191**	**0.057**	–	0.6	1.5	1.6	2.0	2.6	3.2	4.0	4.4	4.6	5.5	6.9
**S8**	**0.110**	**0.306**	**0.119**	**0.081**	**0.094**	**0.046**	**0.050**	–	0.9	1.1	1.5	2.1	3.2	3.5	3.8	4.1	5.0	6.4
**S9**	**0.080**	**0.268**	**0.152**	**0.132**	**0.119**	**0.113**	**0.113**	**0.033**	–	0.4	0.8	1.3	2.4	2.6	3.0	3.2	4.1	5.5
**S10**	**0.048**	**0.378**	**0.205**	**0.220**	**0.230**	**0.208**	**0.192**	**0.103**	**0.123**	–	0.4	1.0	2.1	2.3	2.7	3.0	3.8	5.3
**S11**	**0.110**	**0.290**	**0.135**	**0.105**	**0.137**	**0.075**	**0.113**	**0.033**	**0.080**	**0.122**	–	0.6	1.7	2.0	2.4	2.6	3.5	4.9
**S12**	**0.105**	**0.314**	**0.143**	**0.120**	**0.163**	**0.083**	**0.128**	**0.037**	**0.079**	**0.129**	**0.064**	–	1.1	1.4	1.9	2.0	2.1	4.4
**S13**	**0.243**	**0.349**	**0.247**	**0.202**	**0.317**	**0.227**	**0.245**	**0.166**	**0.177**	**0.272**	**0.192**	**0.145**	–	0.7	1.1	1.3	2.0	3.6
**S14**	**0.185**	**0.407**	**0.222**	**0.178**	**0.247**	**0.168**	**0.223**	**0.134**	**0.155**	**0.230**	**0.119**	**0.147**	**0.141**	–	0.6	0.6	1.5	3.0
**S15**	**0.293**	**0.454**	**0.285**	**0.215**	**0.315**	**0.278**	**0.270**	**0.190**	**0.185**	**0.306**	**0.213**	**0.208**	**0.276**	**0.259**	–	0.5	1.2	2.6
**S16**	**0.062**	**0.393**	**0.203**	**0.202**	**0.236**	**0.178**	**0.190**	**0.101**	**0.130**	**0.064**	**0.138**	**0.096**	**0.254**	**0.180**	**0.282**	–	0.9	2.4
**S17**	**0.330**	**0.370**	**0.247**	**0.260**	**0.340**	**0.276**	**0.309**	**0.222**	**0.236**	**0.323**	**0.188**	**0.210**	**0.225**	**0.216**	**0.369**	**0.312**	–	1.6
**S18**	**0.265**	**0.364**	**0.176**	**0.149**	**0.253**	**0.166**	**0.187**	**0.131**	**0.172**	**0.268**	**0.114**	**0.172**	**0.179**	**0.060**	**0.274**	**0.229**	**0.224**	–

All comparisons remained significantly different after the Benjamini and Yekutieli correction.

**Fig 2:**
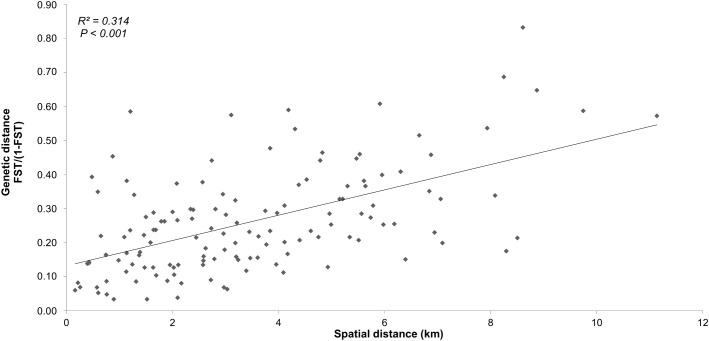
Isolation by distance of *Phelsuma inexpectata* using nuclear markers. The genetic distance (*F*_*ST*_ / (1- *F*_*ST*_)) is plotted against the spatial distance (in km) and is based on the genotyping of 441 specimens at 20 microsatellite markers across 17 sites (excluding site S1) in the south of Reunion Island.

In addition to genetic differentiation between sites, a significant IBD pattern was detected across all 17 sites (excluding the presumably recently introduced site S1) (R^2^ = 0.314, p-value < 0.001) (Fig 2). This pattern remained significant when only natural (8 sites: R^2^ = 0.625, p-value = 0.002) (S2A Fig) or anthropized sites (9 sites, excluding the site S1: R^2^ = 0.402, p-value = 0.012) (S2B Fig) were considered.

### Population clusters and structuration

**Fig 3:**
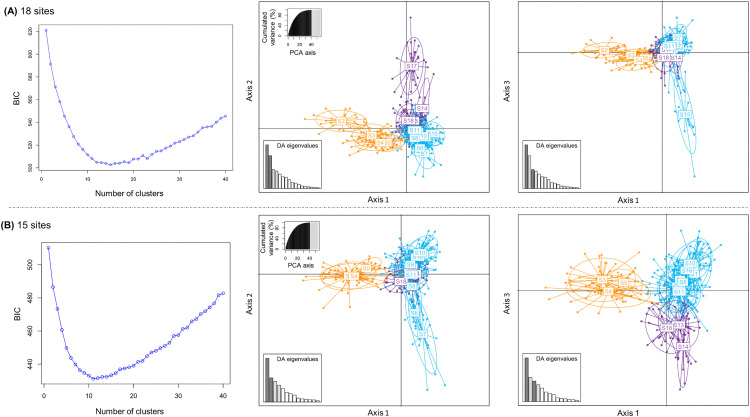
Scatterplots of Discriminant Analysis of Principal Components (DAPC) using 20 microsatellite loci for *Phelsuma inexpectata.* **(A)** DAPC based on 452 geckos from 18 sites. **(B)** DAPC based on 384 geckos from 15 sites (sites S2, S15, and S17 removed). For each analysis, the graphic of BIC plot shows values according to the number of clusters, and scatterplots based on axes 1 and 2, and on axes 1 and 3, are provided. The colors in the scatterplots correspond to those in Fig 1D for *K = 3* (orange, blue, and purple).

The results from STRUCTURE for *K = *1–20 showed that the data are best explained by *K = *3 based on Δ*K* (Fig 1D and S3A Fig). A first genetic cluster (colored in orange in Fig 1D, for *K = *3) comprised four sites: S2 to S5; a second cluster (in blue) comprised ten sites: S1, S6 to S12, S15, and S16; and a third cluster (in purple) comprised four sites: S13, S14, S17, and S18. Globally, this population genetic structure appears to match to a geographical distribution from the west to the east of the study area. However, in some parts this geographical distribution is not perfect, with for instance most individuals from the western site S1 and the eastern sites S15 and S16 being assigned to the central blue cluster. According to the LnP*(K)* method, the data can also be explained by *K* = 12 (Fig 1D and S3B Fig) with some sites forming specific and unique genetic subclusters (see Fig 1D for *K *= 12). This latter result confirmed that sites S1, S10, and S16 belong to the same genetic cluster, and similar findings were also observed for sites S14 and S18 (Fig 1D). The presence of such genetic subclustering pattern is consistent with the fine-scale genetic differentiation pattern between sites (Table 2 and S1 Fig). Results from the DAPC analyses showed similar global patterns, with the three main genetic clusters clearly discriminated (Fig 3). Note that the BIC criterion indicated that the nuclear data were best explained by 15 clusters when all sites were considered (Fig 3A), and by 11 clusters when the most differentiated sites (S2, S15, and S17) were removed from the analyses (Fig 3B).

### Detection of first migrants

Among the 452 individuals, only twelve specimens (2.7%) sampled from 11 sites were considered as first-generation migrants (S3 Table). For eight out of the twelve first-generation migrants, origin sites were geographically close to the sampled sites with distances ranging from 0.2 to 0.6 km. In contrast, origins of the remaining four first-generation migrants were distant from the sampled sites with distances ranging from 1.3 to 9.2 km (S3 Table).

### Detection of bottlenecks

Using the general vertebrate mutation rate, evidence of recent bottlenecks was detected at three sites: S1, S4, and S17. When considering the reptile mutation rate, signs of recent bottlenecks were detected at six sites: S1, S2, S4, S11, S15, and S17 (S4 Table).

## Discussion

In this study, using two mitochondrial and twenty microsatellite loci on 452 geckos sampled across ten anthropized and eight natural sites, we provide the first survey on the genetic diversity and population structure of the Critically Endangered *P. inexpectata.* This species harbors a relatively low genetic diversity compared to other insular members of the *Phelsuma* genus [16,20,68], and shows genetic structuring across its restricted distribution range (i.e., 14 km long) in the south of Reunion Island.

### Low genetic diversity in *Phelsuma inexpectata*

Within the *Phelsuma* genus, native to the Southwestern Indian Ocean islands [31,34], comparable mitochondrial and nuclear genetic studies have shown that genetic estimates differ between species [16,20,68]. To date, *P. inexpectata* displays the lowest mitochondrial and nuclear genetic diversity reported within the *Phelsuma* genus [16,20,68]. Indeed, only nine mitochondrial haplotypes (*cytb* and *12S* combined, 1,190 bp) were found for the Critically Endangered *P. inexpectata* (N = 448 geckos, 18 sites sampled, endemic to Reunion Island) compared to a total of 25 mitochondrial haplotypes (*COI,* 314 bp) for the Least Concern *Phelsuma andamanensis* [69] (N = 123 geckos, 10 islands sampled, endemic to Andaman archipelago) [68], 25 mitochondrial haplotypes (*cytb* and *16S* combined, 777 bp) for the Endangered *Phelsuma guimbeaui* [70] (N = 80 geckos, 13 subpopulations sampled, endemic to Mauritius) [16], and 47 mitochondrial haplotypes (*cytb* and *16S* combined, 1,267 bp) for the Endangered *Phelsuma borbonica* [71] (N = 235 geckos, 19 sites sampled, native to Reunion Island) [20]. Similarly, *N*_*a*_ values from nuclear markers reached a maximum of 2.8 alleles per locus per site for *P. inexpectata* (20 microsatellite markers, N = 452 geckos, 18 sites) compared to a maximum of 15.5 alleles per loci per site for *P. guimbeaui* (20 microsatellite markers, N = 260 geckos, 10 subpopulations) [16]. Both *H*_*o*_ and *H*_*e*_ were also remarkably low, with a maximum value per site of 0.353 and 0.345, respectively, for *P. inexpectata* (20 microsatellite markers, N = 452 geckos, 18 sites), compared to 0.876 and 0.891, respectively, for *P. guimbeaui* (20 microsatellite markers, N = 260 geckos, 10 subpopulations) [16]. For *P. andamanensis,* average *N*_*a*_, *H*_*o*_, and *H*_*e*_ values of 23.7, 0.79, and 0.92 were reported, respectively (13 microsatellite markers, N = 140 geckos, 6 islands) [68]. For *P. borbonica,* the values of *H*_*e*_ per site varied from 0.14 to 0.54 (13 microsatellite markers, N = 235 geckos, 19 sites) [20]. Such differences in genetic diversity may be partly explained by the considerable variation in the species’ respective areas of occupancy. Indeed, *P. inexpectata*, exhibiting the lowest genetic diversity reported so far among the *Phelsuma* genus, occupies a highly restricted area of occupancy of 16 km^2^, which is 10–16 times smaller than that of other species (estimated areas of occupancy of 156 km^2^ and 250 km^2^ for *P. guimbeaui* and *P. borbonica*, respectively) [21,70,71]. Similar low levels of mitochondrial and/or nuclear genetic diversity found here for *P. inexpectata* have also been documented for other threatened reptile species, e.g., *Cyclura cychlura inornata, Gallotia bravoana*, *Gavialis gangeticus,* and *Phyllodactylus sentosus* [14,72–74]. Hence, the genetic diversity harbored by *P. inexpectata* is consistent with the low genetic diversity detected in threatened taxa [75–77]. Such low genetic diversity in *P. inexpectata* could make the species particularly vulnerable to stochastic changes in its environment, in addition to the loss and fragmentation of its habitat.

### Nuclear and mitochondrial genetic diversity in natural and anthropizes sites

Despite the overall low genetic diversity observed for *P. inexpectata* across all sampled sites, both *H*_*o*_ and *H*_*e*_ indices indicate a slightly higher nuclear genetic diversity at natural sites compared to anthropized ones. In addition, mitochondrial genetic diversity at natural sites is remarkable since 56% of the haplotypes were only detected in these sites. Compared to anthropized sites, the natural sites probably harbored more ancient gecko populations, which could be considered as relict populations with the associated original genetic diversity. While these sites were likely part of a more or less continuous natural habitat prior to human arrival on the island, this habitat is now highly fragmented, and the sites are isolated from one another. The available data from medium-term monitoring on sites S6 and S7 indicate clearly that the gecko populations at these sites are declining rapidly over the past decade and that no migration was observed at such fine spatial scale [21,22]. In anthropized sites, genetic diversity was also low and the detection of bottleneck signatures (sites S1, S2, and S15, based on the reptile mutation rate) might reflect either recent introductions or true bottlenecks. Nevertheless, in the absence of knowledge about the historical distribution of *P. inexpectata*, it is not possible to reach clear conclusions. However, available historical aerial photographs and illustrations could provide important information on the establishment and maintenance of some gecko populations. An interesting case is the anthropized site S9, where historical aerial photographs and illustrations show that in the recent past (based on IGN [78] and Indian Ocean historic image library websites [79]), the habitat was clearly unfavorable for the maintenance of a gecko population, as the site consisted mainly of sugar cane plantations. Therefore, the current establishment and maintenance of a gecko population was possible due to the modification of the habitat provided by private gardens and by planting favorable vegetation, such as screwpine (*P. utilis*), which largely serves as a windbreak along the coastline [80]. In addition, at site S9, the low mitochondrial haplotype diversity (N = 3) detected, despite the high number of examined specimens (N = 70 geckos), supports the conclusion that the current gecko population results from recent colonization events. Hence, studies based on historical data could provide information on the current distribution of *P. inexpectata*, giving insights into colonization events and the evolution of habitats. Importantly, the presence of *P. inexpectata* in anthropized sites shows the adaptability of the species to human development given the availability of favorable conditions through adapted vegetation [23].

### Fine-scale genetic differentiation of *Phelsuma inexpectata* populations and global genetic structure

Within its limited range, *P. inexpectata* exhibits a fine-scale geographical genetic differentiation, with a global genetic structure that aligns with the geographical distribution of sites. Indeed, the genetic structure reveals distinct clusters globally arranged along an east-west gradient, as well as the IBD patterns detected. The overall genetic structure (for *K = *3) may reflect a historically more or less continuous distribution, where natural physical barriers such as ravines, could have limited gecko dispersal. More recently, anthropogenic barriers such as unsuitable agricultural landscapes may have disrupted gene flow, thereby increasing the isolation of certain sites. The limited dispersal capacity reported for *P. inexpectata*, with a maximum dispersal distance of 100 m [24], may also contribute to the observed genetic structure and particularly the IBD patterns. Lastly, another hypothesis to explain this fine-scale genetic differentiation is the laying site fidelity of females reported for some gecko populations [28]. Based on the nuclear data, the Bayesian STRUCTURE analyses also showed the presence of genetic subclusters (for *K = *12). This genetic substructure and differentiation could also be explained by the factors mentioned above and notably the low migration rates of geckos between sites supported by the identification of only 12 geckos (2.7% of the sampled geckos) as first-generation migrants in our analyses. Interestingly, the presence of geckos belonging to unexpected genetic clusters in some parts of the geographical distribution (examples: site S1 at the extreme west and sites S15 and S16 in the eastern part of the study area; see Fig 1D for *K = *3) highlights again the impact of anthropogenic activities on the shaping of *P. inexpectata* genetic structure. Indeed, these unexpected genetic clusters probably result from introductions of geckos to new areas by active or passive transport of individuals (adults, juveniles or eggs) *via* vehicles and/or plants as reported for *P. inexpectata* but also other *Phelsuma* species on the island [24,29,81]. This is very likely the case at site S1 where geckos were presumably recently introduced, and it is probably the case of several other anthropized sites. In summary, in the light of our results, the current distribution and structuring of the genetic diversity of *P. inexpectata* likely result from different non-exclusive processes, such as historical geographical distribution, limited dispersal of geckos, anthropogenic activities (with fragmentation and isolation of natural populations), and the colonization of novel areas (often urban) through intentional or unintentional transportation of geckos over time.

## Conclusions

The present study constitutes the first overview of the genetic diversity and population structure of the Critically Endangered *P. inexpectata* on Reunion Island. The main findings allow us to provide recommendations for the conservation of the species. Firstly, the detected fine-scale population structuring coupled with the low dispersal capacity of *P. inexpectata* call for attention to the potential local gecko population extinctions in the context of habitat fragmentation and loss. Considering that the geographic distances between most sites exceed the species’ dispersal capacity, and given the potential risk of invasion by non-native gecko species [23] the establishment of corridors at such spatial scales is unlikely to represent an immediate conservation priority, although this may warrant reconsideration under changing future conditions and a site-by-site examination. On the other hand, priority should be given to preventing further loss of suitable habitats for the gecko and, whenever possible, to restoring degraded sites such as the habitat restoration actions ongoing at sites S6 and S7. Secondly, the relatively low genetic diversity of *P. inexpectata* could limit its evolutionary potential and make the species vulnerable to stochastic changes in its environment. Therefore, it is important to conserve the current genetic diversity notably by conserving gecko populations from natural sites harboring original and remarkable genetic diversity (e.g., sites S3, S8 or S12). Although remaining particularly vulnerable to intense and multiple anthropogenic pressures, anthropized sites should also be integrated in conservation programs as they can maintain gecko populations and harbor a specific genetic diversity (e.g., sites S9, S10 or S15). Finally, we stress the importance of carrying out more studies to clearly identify the ecological factors (e.g., demography, behavior, presence of invasive species etc.) underlying the decline of the *P. inexpectata* populations. Pending a better understanding of the mechanisms at play at each site and the implementation of targeted management actions, *ex situ* actions such as breeding program [39] should be considered.

## Supporting information

S1 Fig UPGMA tree of 18 sites of *Phelsuma inexpectata.* Genetic distance was calculated by using pairwise *F*_*ST*_ measures of genetic distance.
(TIF)

S2 Fig Isolation by distance (IBD) (genetic distance *F*_*ST*_ / (1-*F*_*ST*_) *vs* spatial distance between sites in km) of *Phelsuma inexpectata* genotyped at 20 microsatellite markers. (A) IBD at eight natural sites (N_ind_ = 201) and (B) nine anthropized sites (N_ind_ = 240) in the south of Reunion Island.
(TIF)

S3 Fig Graphs of (A) *ΔK* and (B) LnP(K) for *K = *1–20. The results were generated from Bayesian analyses with STRUCTURE based on 452 *Phelsuma inexpectata* genotyped at 20 microsatellite markers.
(TIF)

S1 Table Number of *Phelsuma inexpectata* specimens sampled at 18 sites (S1 to S18) by to sex (females, males, and undetermined: sex under.). For each site, the type of habitat is provided, A: anthropized; N: natural.
(DOCX)

S2 Table Characteristics of the 20 microsatellite loci used for the 452 *Phelsuma inexpectata* sampled at 18 sites on Reunion Island. *Na*_*tot*_ total number of alleles detected; *H*_*o*_ the observed heterozygosity (mean over sites), *H*_*e*_ the expected heterozygosity; *Null allele detected* detection of null allele and the associated sites. All loci were in Hardy–Weinberg equilibrium after Benjamini and Yekutieli corrections.
(DOCX)

S3 Table First-generation migrants based on 20 microsatellite markers. The identification number and the sex of specimens are provided. The probability thresholds, the sampled site, putative origin site, and the distance between sampled and putative origin sites are also provided.
(DOCX)

S4 Table One-tailed Wilcoxon signed-rank test results obtained from BOTTLENECK software under the Two-Phase Mutation Model with different percentages of SMM and variance (30 or 12%). Bold values indicate statistical significance at p-value < 0.05 for the one-tailed Wilcoxon sign-rank test.
(DOCX)
