# Supplementary material for: First evaluation of genetic diversity and population structure of Phelsuma inexpectata (Gekkonidae), a critically endangered gecko endemic to Reunion Island
Source: PLoS One. 2025 Dec 12;20(12):e0338217. doi: 10.1371/journal.pone.0338217 (PMC12700416; doi:10.1371/journal.pone.0338217)
Supplement: S1 Table — For each site, the type of habitat is provided, A: anthropized; N: natural. (DOCX) [file pone.0338217.s004.docx]

**S1 Table. Number of *Phelsuma inexpectata* specimens sampled at 18 sites (S1 to S18) by sex (females, males, and undetermined: sex under.).**

| **Site** | **Habitat** | **N total** | **N ♀** | **N ♂** | **N sex under.** |
| --- | --- | --- | --- | --- | --- |
| S1 | A | 11 | 3 | 8 | - |
| S2 | A | 23 | 9 | 14 | - |
| S3 | N | 32 | 14 | 18 | - |
| S4 | N | 30 | 17 | 13 | - |
| S5 | A | 15 | 4 | 11 | - |
| S6 | N | 13 | 11 | 2 | - |
| S7 | N | 19 | 3 | 14 | 2 |
| S8 | N | 17 | 9 | 8 | - |
| S9 | A | 71 | 34 | 36 | 1 |
| S10 | A | 29 | 10 | 18 | 1 |
| S11 | N | 30 | 15 | 15 | - |
| S12 | N | 30 | 16 | 14 | - |
| S13 | A | 30 | 12 | 18 | - |
| S14 | A | 12 | 1 | 11 | - |
| S15 | A | 15 | 4 | 11 | - |
| S16 | A | 20 | 6 | 13 | 1 |
| S17 | N | 30 | 16 | 14 | - |
| S18 | A | 25 | 14 | 11 | - |
| *Total* | | 452 | 198 | 249 | 5 |
|  |  |  |  |  |  |
| For each site, the type of habitat is provided, A: anthropized; N: natural | | | | | |
