# Supplementary material for: First evaluation of genetic diversity and population structure of Phelsuma inexpectata (Gekkonidae), a critically endangered gecko endemic to Reunion Island
Source: PLoS One. 2025 Dec 12;20(12):e0338217. doi: 10.1371/journal.pone.0338217 (PMC12700416; doi:10.1371/journal.pone.0338217)
Supplement: S2 Table — Natot total number of alleles detected; Ho the observed heterozygosity (mean over sites), He the expected heterozygosity; Null allele detected detection of null allele and the associated sites. All loci were in Hardy–Weinberg equilibrium after Benjamini and Yekutieli corrections. (DOCX) [file pone.0338217.s005.docx]

**S2 Table. Characteristics of the 20 microsatellite loci used for the 452 *Phelsuma inexpectata* sampled at 18 sites on Reunion Island.**

| **Locus** | **Genbank  Acc . No.** | **Repeat motif** | **Observed size range (bp)** | **No. of sequenced specimens (amplification rate %)** | ***Na_tot_*** | ***H_o_*   (mean over pop)** | ***H_e_*  (mean over pop)** | **Null allele  detected** |
| --- | --- | --- | --- | --- | --- | --- | --- | --- |
| **Pinex_06** | OP556336 | (GA)11 | 232 and 234 | 451 (99.8%) | 2 | 0.179 | 0.182 | no |
| **Pinex_07** | OP556337 | (GA)11 | 205 - 221 | 436 (96.5%) | 7 | 0.304 | 0.310 | no |
| **Pinex_11** | OP556338 | (AATG)9 | 189 - 201 | 432 (95.6%) | 4 | 0.517 | 0.520 | no |
| **Pinex_15** | OP556339 | (GTTT)7 | 295 and 299 | 451 (99.8%) | 2 | 0.198 | 0.195 | no |
| **Pinex_22** | OP556340 | (AC)6 | 293 - 297 | 387 (85.6%) | 3 | 0.435 | 0.423 | yes in S4 site |
| **Pinex_23** | OP556341 | (AG)6 | 252 - 256 | 434 (96.0%) | 3 | 0.189 | 0.192 | no |
| **Pinex_26** | OP556342 | (TC)17 | 134 - 146 | 423 (93.6%) | 6 | 0.332 | 0.353 | no |
| **Pinex_33** | OP556343 | (AG)10 | 215 - 226 | 414 (91.6%) | 4 | 0.252 | 0.224 | no |
| **Pinex_34** | OP556344 | (AC)10 | 200 - 208 | 450 (99.6%) | 5 | 0.284 | 0.263 | no |
| **Pinex_35** | OP556345 | (GA)10 | 199 and 201 | 443 (98.0%) | 2 | 0.350 | 0.351 | no |
| **Pinex_44** | OP556346 | (TG)9 | 163 and 165 | 446 (98.7%) | 2 | 0.081 | 0.098 | yes in S13 site |
| **Pinex_46** | OP556347 | (AT)9 | 149 - 165 | 397 (87.8%) | 7 | 0.496 | 0.494 | yes in S12 and S18 sites |
| **Pinex_47** | OP556348 | (TC)9 | 128 - 136 | 433 (95.8%) | 3 | 0.300 | 0.323 | no |
| **Pinex_52** | OP556349 | (TG)8 | 205 and 209 | 447 (98.9%) | 2 | 0.023 | 0.022 | no |
| **Pinex_58** | OP556350 | (CT)8 | 157 and 159 | 432 (95.6%) | 2 | 0.197 | 0.171 | no |
| **Pinex_61** | OP556351 | (TG)7 | 295 - 303 | 419 (92.7%) | 4 | 0.387 | 0.351 | yes in S3 site |
| **Pinex_62** | OP556352 | (AATC)7 | 285 - 309 | 395 (87.4%) | 6 | 0.316 | 0.331 | yes in S6 site |
| **Pinex_72** | OP556353 | (GT)7 | 145 - 151 | 429 (94.9%) | 3 | 0.052 | 0.049 | no |
| **Pinex_87** | OP556354 | (AC)6 | 175 - 183 | 422 (93.4%) | 3 | 0.127 | 0.130 | no |
| **Pinex_88** | OP556355 | (TTCA)6 | 156 - 172 | 441 (97.6%) | 5 | 0.493 | 0.478 | no |

*Na_tot_* total number of alleles detected; *H_o_* the observed heterozygosity (mean over sites), *H_e_* the expected heterozygosity; *Null allele detected* detection of null allele and the associated sites. All loci were in Hardy–Weinberg equilibrium after Benjamini and Yekutieli corrections.
