# Supplementary material for: First evaluation of genetic diversity and population structure of Phelsuma inexpectata (Gekkonidae), a critically endangered gecko endemic to Reunion Island
Source: PLoS One. 2025 Dec 12;20(12):e0338217. doi: 10.1371/journal.pone.0338217 (PMC12700416; doi:10.1371/journal.pone.0338217)
Supplement: S3 Table — The identification number and the sex of specimens are provided. The probability thresholds, the sampled site, putative origin site, and the distance between sampled and putative origin sites are also provided. (DOCX) [file pone.0338217.s006.docx]

**S3 Table. First-generation migrants based on 20 microsatellite markers.** The identification number and the sex of specimens are provided. The probability thresholds, the sampled site, putative origin site, and the distance between sampled and putative origin sites are also provided.

| **Specimen** | **Sex** | **Sampled site** | **Originated site** | **Distance between sites  (km)** | **Probability threshold** |
| --- | --- | --- | --- | --- | --- |
| n°094 | ♂ | S1 | S10 | 9.2 | 0.008 |
| n°346 | ♂ | S3 | S4 | 0.2 | 0.001 |
| n°180 | ♀ | S4 | S5 | 0.3 | 0.007 |
| n°412 | ♂ | S5 | S4 | 0.3 | 0.001 |
| n°413 | ♀ | S5 | S4 | 0.3 | 0.009 |
| n°330 | ♂ | S7 | S8 | 0.6 | 0.006 |
| n°321 | ♀ | S9 | S10 | 0.4 | 0.004 |
| n°081 | ♀ | S11 | S12 | 0.6 | 0.000 |
| n°138 | ♀ | S12 | S11 | 0.6 | 0.005 |
| n°058 | ♂ | S16 | S13 | 1.3 | 0.000 |
| n°236 | ♂ | S17 | S13 | 2.0 | 0.001 |
| n°113 | ♀ | S18 | S7 | 6.9 | 0.002 |
