# Supplementary material for: First evaluation of genetic diversity and population structure of Phelsuma inexpectata (Gekkonidae), a critically endangered gecko endemic to Reunion Island
Source: PLoS One. 2025 Dec 12;20(12):e0338217. doi: 10.1371/journal.pone.0338217 (PMC12700416; doi:10.1371/journal.pone.0338217)
Supplement: S4 Table — Bold values indicate statistical significance at p-value < 0.05 for the one-tailed Wilcoxon sign-rank test. (DOCX) [file pone.0338217.s007.docx]

**S4 Table. One-tailed Wilcoxon signed-rank test results obtained from BOTTLENECK software under the Two-Phase Mutation Model with different percentages of SMM and variance (30% or 12%).** Bold values indicate statistical significance at p-value < 0.05 for the one-tailed Wilcoxon signed-rank test.

| **Site** | **78% of SMM (vertebrate)** | | **54% of SMM (reptile)** | |
| --- | --- | --- | --- | --- |
|  | 30% of variance | 12% of variance | 30% of variance | 12% of variance |
| S1 | **0.000732** | **0.000732** | **0.000732** | **0.000732** |
| S2 | 0.050781 | 0.061523 | **0.041504** | **0.041504** |
| S3 | 0.105713 | 0.105713 | 0.064865 | 0.071930 |
| S4 | **0.002579** | **0.005493** | **0.000420** | **0.000420** |
| S5 | 0.244354 | 0.262238 | 0.165131 | 0.179565 |
| S6 | 0.380768 | 0.423462 | 0.339386 | 0.339386 |
| S7 | 0.661148 | 0.644051 | 0.626671 | 0.626671 |
| S8 | 0.573181 | 0.626671 | 0.426819 | 0.444984 |
| S9 | 0.132263 | 0.132263 | 0.054192 | 0.054192 |
| S10 | 0.876896 | 0.901806 | 0.826725 | 0.837635 |
| S11 | 0.071930 | 0.079529 | **0.041626** | 0.052292 |
| S12 | 0.216599 | 0.247711 | 0.087677 | 0.105713 |
| S13 | 0.133789 | 0.133789 | 0.059448 | 0.086304 |
| S14 | 0.596130 | 0.596130 | 0.291504 | 0.380432 |
| S15 | 0.075684 | 0.075684 | **0.038574** | **0.046143** |
| S16 | 0.380768 | 0.401978 | 0.194702 | 0.299744 |
| S17 | **0.028992** | **0.039246** | **0.017639** | **0.017639** |
| S18 | 0.576538 | 0.576538 | 0.511017 | 0.554810 |
